# Enhancing Metagenomics Investigations of Microbial Interactions with Biofilm Technology

**DOI:** 10.3390/ijms141122246

**Published:** 2013-11-11

**Authors:** Robert J. C. McLean, Kavita S. Kakirde

**Affiliations:** Department of Biology, Texas State University, 601 University Drive, San Marcos, TX 78666, USA

**Keywords:** biofilm-enrichment, metagenomics, functional screening, sequence-based screening, microbial interactions

## Abstract

Investigations of microbial ecology and diversity have been greatly enhanced by the application of culture-independent techniques. One such approach, metagenomics, involves sample collections from soil, water, and other environments. Extracted nucleic acids from bulk environmental samples are sequenced and analyzed, which allows microbial interactions to be inferred on the basis of bioinformatics calculations. In most environments, microbial interactions occur predominately in surface-adherent, biofilm communities. In this review, we address metagenomics sampling and biofilm biology, and propose an experimental strategy whereby the resolving power of metagenomics can be enhanced by incorporating a biofilm-enrichment step during sample acquisition.

## 1. Introduction

The global distribution of microorganisms is impressive, ranging from the deep subsurface in terrestrial [1] and marine environments [2], to the upper atmosphere [3]. Although culturing techniques are improving, the vast majority of microorganisms in natural environments including soil are as yet uncultured. Estimates of microbial composition, diversity, and even ecological interactions are performed using a variety of culture-independent approaches including metagenomics [4]. One highly notable early achievement from molecular investigations was the identification of three domains of life, Archaea, Bacteria, and Eukarya [5]. The advances of sequencing technology from the traditional Sanger protocol to higher throughput, more economical approaches such as pyrosequencing and Illumina-based sequencing [6] have resulted in the generation of considerable data, and as a result these systems biology approaches require considerable bioinformatics analysis and genome sequence construction [7]. A number of highly significant results have arisen from metagenomics studies including the discovery of “Candidatus *Pelagibacter ubique*” strain HTCC1062, originally identified as clade SAR11, which is considered the most abundant microorganism in the pelagic ocean [8]. Based on genome analysis, unusual nutrient requirements for “Ca. *P. ubique*” were identified and this extreme oligotroph can now be cultured on defined media [9].

## 2. Experimental Strategies for Extraction of Metagenomic DNA from Soil Biofilms

Surface-adherent microbial communities (biofilms) are a common feature of microbial growth in many environments [10] including soils. In the investigation of a soil biofilm it may be of particular interest to look at specific sections that may indicate a multitude of interactions between microbial populations in the biofilm. Visualization and imaging using microscopy techniques can be used to target this subset of the entire microbial population from the sample biofilm. There are two methods for the extraction and processing of metagenomic DNA from a microbial population, direct and indirect extraction. In the direct extraction method pioneered by Ogram *et al.* [11], any extracellular DNA is first separated from the environmental sample by treating it with an alkaline buffer. The cells in the matrix are then subjected to direct mechanical (e.g., bead beating) lysis followed by extraction of DNA released from these cells. DNA recovered by centrifugation is then concentrated and purified before cloning. In contrast, the indirect method involves recovery of microbial cells from the sample. The recovered cells are subjected to cell lysis (chemical and enzymatic) followed by DNA extraction and purification [12]. Although time-consuming the indirect extraction method prevents the contamination from non-bacterial DNA [13] that may be present in the sample. Direct extraction methods provide high yield of lower size DNA fragments whereas indirect methods provide low yield of higher size DNA fragments. Both methods have distinct advantages and limitations, and the choice should be based on the intended downstream application and the objective of the study. Irrespective of the DNA extraction method, care must be taken to avoid co-isolation of organic compounds that may be present in the sample and can inhibit downstream processes. Various factors to be considered pertaining to soil metagenomics and the use of specific strategies based on the ultimate goal of the study are discussed by Kakirde *et al.* [14] and this provides a good guideline for designing a metagenomics project. Since there are multiple approaches that can be adopted at each stage of a metagenomic analysis it is important to select appropriate DNA extraction and purification methods and consider if cloning is necessary.

Direct sequencing of metagenomic DNA can be performed followed by sequence analysis. The vastly growing field of next generation sequencing technology offers a plethora of options for sequencing such as 454 Pyrosequencing and Illumina among others. Every platform offers different coverage and read length and the cost per base of sequencing is likely to become more affordable with the rapid advances in this field. The massive amount of sequence data generated by next-generation sequencers requires the use of specialized bioinformatics tools to mine and analyze the output. The sequence-only method is comparatively less time-consuming than the alternative, which is construction of metagenomic libraries and subsequent function and or sequence-based screening to identify gene products encoded by the target microbial partners. An appropriate cloning vector and a host organism should be used in capturing and cloning these genes. Depending on the desired insert size and purity, the DNA for cloning in many instances can be obtained by using commercially available kits (such as Qiagen and MoBio). Some of the methods commonly used for purification of extracted DNA are the standard phenol-chloroform extraction, cesium chloride density gradient centrifugation and chromatography. Often a combination of methods can lead to greater purity but this is also accompanied by increased DNA loss. Hence the purification protocol(s) should be selected according to the requirements of the concentration and purity of the DNA that is to be cloned. Prior to cloning DNA can be sheared using physical shearing or partial restriction digestion, size-selected by electrophoresis [15] and then electroeluted [16]. Cosmid and fosmid vectors have been used for cloning DNA from environmental samples with an insert size between 30 and 50 kb [14]. Fosmids are based on the bacterial F-factor and are stably maintained in the host due to their low copy number (1–2 copies per cell), which is tightly regulated in a host such as *E. coli*. Fosmid vectors have a higher cloning efficiency as compared to bacterial artificial chromosome (BAC) vectors. A limitation of fosmid vectors is the limited insert size. Larger inserts can be cloned by using a BAC vector, which can easily maintain fragments greater than 100 kb [17]. BAC vectors can be induced to a high copy number for increased expression and DNA yield from metagenomic clones, and can also be stably maintained at single copy [18]. In investigating specific interactions within the biofilm such as syntrophy, competition or the transfer of antibiotic resistance elements cloning would be preferable to the sequence only approach especially when looking for novel mechanisms. *E. coli* is one of the commonly used heterologous hosts in construction of metagenomic libraries since it has a high cloning efficiency and is easy to culture and work with *in vitro* [19–22]. Other heterologous hosts such as *Streptomyces* species have been used for heterologous expression of cloned metagenomic DNA in multiple studies [23,24]. The use of Archaea, specifically extreme halophiles as a host for expression of cloned DNA has been done in previous studies. The percent G + C content of the cloned genes, predominant partners (Gram positive or Gram negative) in the biofilm samples are some factors that can be considered in selecting a suitable host. Vectors systems used in the process should also be compatible with the selected host organism.

**Figure 1:**
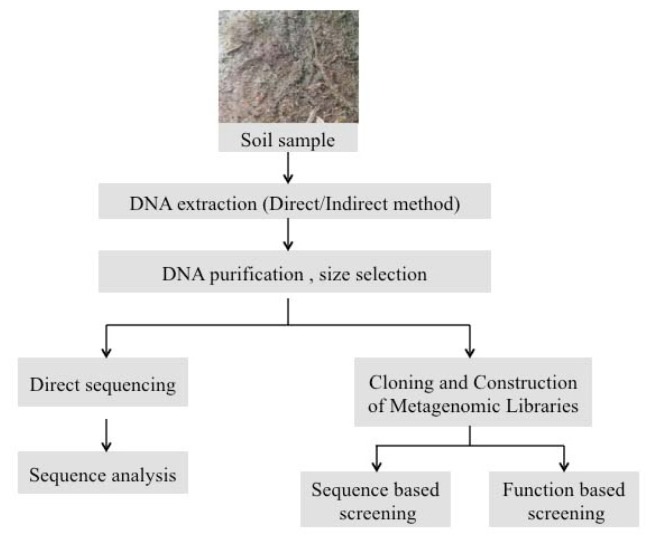
General steps in a metagenomics strategy to investigate microbial communities in environmental samples.

Construction of metagenomic libraries followed by a function-based screening is an excellent strategy to actually detect the gene products of the cloned inserts and could be used to identify various metabolic products, including both growth enhancing as well as antimicrobial compounds produced by microbial partners in the biofilm. The effect of these compounds on various tester microorganisms can be determined by using a bioassay method in the functional screen. Similarly the presence of specific antimicrobial resistance elements can be detected by incorporating the particular antibiotic in the bioassay during screening of the metagenomic clones. Although cost-intensive, if feasible a combined sequence and function based analysis can be very effective in determining the chemistry and basic charcteristics of the microbial partners in the biofilm interaction. The preliminary information obtained from the sequence data can be used for designing a specifically targeted function based metagenomics screen. Figure 1 summarizes the general steps of a metagenomics strategy to investigate microbial communities in environmental samples.

In addition to identifying genes of interest, a sequence based screening of the metagenomic libraries can be used in identification of regulatory elements that have been shown to control the formation and structure of biofilms [25]. A sequence only approach utilizing the power of the 454 sequencing technology is a good strategy for this purpose and yields good quality metagenomic sequences. These sequences can be deposited in GenBank and then referenced against available environmental databases and metagenomic datasets. The metagenomics RAST (MG-RAST) server is an excellent and free public resource that compares both protein and nucleotide databases to generate phylogenetic and functional summaries of the metagenomic sequence data [26]. MEGAN (Metagenome Analyzer), a computer program is another bioinformatics tool for analysis of high-throughput metagenomic sequence data and gene prediction that compares DNA reads against databases using comparative tools such as BLAST [27]. Metagenomic sequence analysis of microbial communities in a biofilm using the tools mentioned here can be used to identify and predict gene functions and can provide a different perspective to investigate the dynamic interactions between microbial partners within the biofilm environment.

## 3. Bacterial Adhesion and Biofilm Ecology

Bacterial adhesion to surfaces has been known for some time [28] but has only been recognized as a dominant mode of bacterial growth in nature in the past 20–30 years [10,29]. Surface-adherent microbial communities, now referred to as biofilms [10] are common in most environments. The prominence of biofilms is easily explained in flowing systems such as rivers [30] or pipelines [31], wherein surface adhesion enables microorganisms to persevere in spite of shear forces. Nutrients adsorb onto surfaces and microorganisms would therefore be attracted to sources of nutrition—a phenomenon sometimes referred to as the bottle effect [32]. Metabolic and genetic interactions are facilitated when organisms grow in close proximity within biofilms. Wolfaardt *et al.* [33] studied the ability of soil bacteria to grow on a commercial herbicide, diclophop methyl and found that bacteria could survive on this compound as a sole carbon source only if present as a biofilm consortium. Pure cultures of the soil isolates were unable to grow on this herbicide regardless of whether they were grown as planktonic or biofilm cultures. Similarly, mixed planktonic cultures were unable to grow on this herbicide [33]. Nitrification is another well-known biological phenomenon consisting of a two step process involving ammonia oxidation to nitrite, followed by nitrite oxidation to nitrate [34]. Ammonia oxidizing microorganisms are found in close proximity to nitrite oxidizers within nitrifying biofilms [35,36]. Syntrophic metabolism within microbial aggregates has also been reported in interspecies hydrogen transfer during anaerobic digestion of cellulose [37,38]. Biofilm growth has also been shown to promote genetic exchange through transformation [39] and conjugation [40,41] due to the close proximity of the donor and recipient organisms.

Biofilm studies with pure cultures have shown that these communities go through a developmental process [42] involving initial adhesion of microorganisms to a surface, aggregation into clumps (microcolonies), a maturation process and finally a dispersion process. In some organisms, notably *Pseudomonas aeruginosa*, *Staphylococcus aureus* and *Vibrio cholerae*, genes and mechanisms for biofilm development have been identified (reviewed in [42–44]). At the morphological level, there is evidence that similar processes occurs within mixed community biofilms, with the added complication of ecological interactions between species. In the dental field, there has been considerable work showing the population development of biofilms on teeth (dental plaque). When a hydroxyapatite tooth surface is cleaned, it becomes rapidly coated by adsorbed salivary proteins, which form a conditioning film [45]. Primary colonizing bacteria including *Streptococcus gordonii*, *Streptococcus oralis* and *Actinomyces naislundii* then attach to the conditioning film [46] and are in turn colonized by other organisms such as the cariogenic gram positive *Streptococcus mutans* [47]. Cell surface features including surface carbohydrates and carbohydrate-binding proteins (lectins), permitting the binding (coaggregation) of individual species to each other, is a major feature of population development in dental biofilms [47]. Microbial succession certainly occurs in other environments [48–50], and in biofilms associated with higher organisms, the host may play an active role in biofilm development. In the rhizosphere, plant exudates function as bacterial nutrients and play an important role in bacterial recruitment, and associated biofilm development and bacterial succession [50]. Cell signal interactions [51–53] are also important, during microbial colonization, biofilm formation and population succession. Other factors that are also important during biofilm population development include antimicrobial vesicle formation [54], antimicrobial chemicals [55] and bacteriocins [56]. At least two studies have shown that polymicrobial biofilms are more resistant to antibacterial agents and stress, than single species biofilms [57,58].

**Figure 2:**
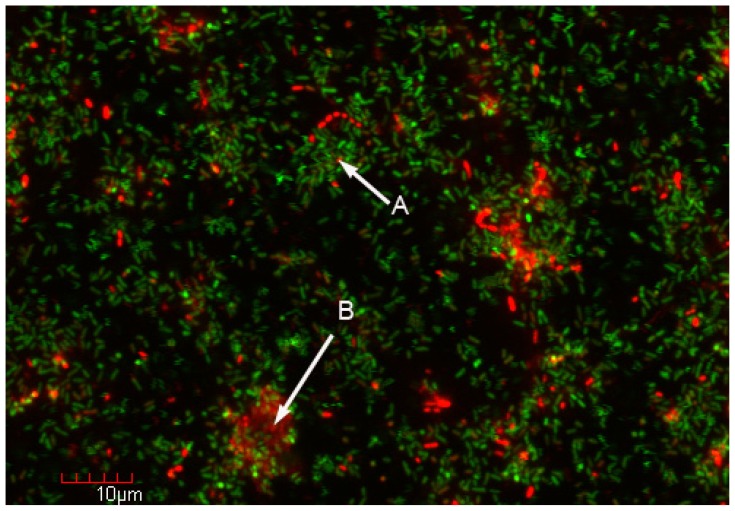
Biofilm enrichment strategy for metagenomics investigation. Confocal microscopy examination of a mixed population biofilm of *E. coli* and *P. aeruginosa* stained with the Live/Dead™ stain (Life Technologies, Grand Island, NY, USA) reveals microcolonies with viable (**A**) and non-viable (**B**) cells. Sampling and metagenomics analyses from these two microcolonies could suggest mechanisms underlying the loss of viability or other cellular interactions. While conventional genetic analyses can be performed for a mixed population biofilm containing known, genetically tractable organisms such as *E. coli* and *P. aeruginosa* [62], it is not practical for many naturally occurring biofilms with potentially unculturable organisms. This combination biofilm-enrichment strategy for metagenomics would be particularly useful in natural biofilms wherein the component populations may not be known.

Another feature of biofilms is an indication of cell specialization. This is particularly prominent and well-described in biofilms formed by the social bacterium, *Myxococcus xanthus* in which some cells are involved in reproduction, others in nutrient acquisition, and others have structural roles [59]. Similar analogies have been shown in other organisms [43]. Certainly chemical gradients including nutrient levels, pH, and oxygen levels (in aerobic biofilms) result in a physiological gradient [60]. The structure and specialization seen within biofilms has been likened to a city [61] (Figure 2), with different physiological functions and even component species being present in clusters (microcolonies). Using the city metaphor for biofilms [60], an individual microcolony may function as one apartment building and will have ecological interactions (synergy, antagonism, synthrophic metabolism, genetic exchange, *etc.*) with neighboring microcolonies (“apartment buildings”). While biofilm structure and function is certainly complex, it largely reflects the situation in which bacteria naturally exist. As a result, broad based molecular microbial ecology studies would benefit by focusing on biofilms.

## 4. Biofilm Technology and Its Potential Application to Molecular Microbial Ecology

In most environments, microorganisms live as surface-adherent biofilm communities [10]. Within biofilms, many and possibly most microbial interactions and processes occur. Included in naturally occurring biofilm communities are cultivable and non-cultivable microorganisms [4]. While broad-based molecular approaches, such as metagenomics offer an invaluable insight to identifying new organisms and potential interactions, the methods commonly used to obtain the genetic material obtain samples from relatively large samples and as a result data and interpretations are based on sample averaging, which would include biofilm and planktonic populations, and likely cellular fragments and extracellular DNA. As shown in Figure 2, we propose the incorporation of biofilm technology as an experimental strategy to obtain higher resolution and more accurate investigations of microbial activities and interactions as they occur in nature.

The ideal strategy to study biofilms would be to examine samples *in situ* or alternatively those obtained directly from the field (or host if associated with a higher organism). Except for the molecular approaches used, this strategy mimics the direct morphological examinations of biofilms performed by Zobell [28], Costerton [10] and others. In the case of easily obtained and accessible biofilms such as those associated with rock surfaces in streams ([63] or urinary catheter infections [64], access to biofilms is not an issue. Problems arise with inaccessible biofilms, particularly if these biofilms occur in the deep subsurface [2,65], or alternatively with water circulating systems in nuclear facilities [66]. While practical aspects of biofilm accessibility and data reproducibility are certainly considerations in natural samples, experimental manipulation may not be feasible. To circumvent this, a number of sampling protocols have been developed for the study of biofilms. At the simplest level, glass microscope slides or other suitable substrata may be inserted into water or soil and will be readily colonized by resident bacteria [67]. Alternatively, liquid from a pipeline or cooling system can be diverted through a biofilm sampling device [31]. An excellent three volume set of *Methods in Enzymology* [68–70] was published in 1999 and 2001, which summarizes many commonly used techniques used for biofilm research. As well, standardized biofilm growth and testing protocols for antimicrobial agent susceptibility have been developed [71–73].

As stated earlier, biofilm structure is complex and many physiological activities may change from one small population of cells (consortia) to another. Ideally, broad-based metagenomics processes to identify organisms and genes, as well as other complementary approaches such as RNA-seq [74], metabolomics [75] and proteomics [76] approaches to identify gene expression and microbial activity, could be mapped at the single cell level or within small consortia. The biofilm enrichment process for metagenomics is shown in Figure 2. Given the low (typically sub fmole) concentration of molecules in bacteria [77], analytical methods and detection limits need to be refined. As an alternative approach, broad based approaches could be used on whole biofilms and then reporter genes and chemically sensitive probes could be used to map activity using confocal microscopy [60,78]. Several fundamentally important biological issues could be addressed by this biofilm-enrichment metagenomics strategy including the mechanisms whereby microbial interactions occur in nature, do novel unrecognized interactions occur, do previously unknown organisms participate, and finally where do these interactions occur.

## 5. Conclusions

Direct observations of most natural environments reveal that microorganisms frequently exist within surface-adherent biofilm communities [10,43,47]. Similarly, the majority of organisms in many environments cannot be cultured but are identified through culture-independent techniques including metagenomics [3,4,6,19]. Aside from the identification of community members, culture-independent techniques are used to infer microbial interactions [58]. A number of studies using reporter gene technology and confocal microscopy reveal microbial interactions including genetic exchange, signaling, and metabolite exchange to occur between adjacent microorganisms within biofilm communities [34,36,78]. Here, we propose the use of biofilm-enrichment as an experimental strategy to enhance the resolving power of metagenomics and other culture-independent techniques to identify novel microbial interaction mechanisms.
